# Vorinostat in solid and hematologic malignancies

**DOI:** 10.1186/1756-8722-2-31

**Published:** 2009-07-27

**Authors:** David Siegel, Mohamad Hussein, Chandra Belani, Francisco Robert, Evanthia Galanis, Victoria M Richon, José Garcia-Vargas, Cesar Sanz-Rodriguez, Syed Rizvi

**Affiliations:** 1 Hackensack University Medical Center, Hackensack, NJ, USA; 2 H. Lee Moffitt Cancer Center, Tampa, FL, USA; 3 Penn State Cancer Institute, Hershey, PA, USA; 4 University of Alabama, Birmingham, AL, USA; 5 Mayo Clinic College of Medicine, Rochester, MN, USA; 6 Merck Research Laboratories, Upper Gwynedd, PA, USA; 7 Merck Research Laboratories, Madrid, Spain

## Abstract

Vorinostat (Zolinza^®^), a histone deacetylase inhibitor, was approved by the US Food and Drug Administration in October 2006 for the treatment of cutaneous manifestations in patients with cutaneous T-cell lymphoma who have progressive, persistent or recurrent disease on or following two systemic therapies. This review summarizes evidence on the use of vorinostat in solid and hematologic malignancies and collated tolerability data from the vorinostat clinical trial program. Pooled vorinostat clinical trial data from 498 patients with solid or hematologic malignancies show that vorinostat was well tolerated as monotherapy or combination therapy. The most commonly reported drug-related adverse events (AEs) associated with monotherapy (*n *= 341) were fatigue (61.9%), nausea (55.7%), diarrhea (49.3%), anorexia (48.1%), and vomiting (32.8%), and Grade 3/4 drug-related AEs included fatigue (12.0%), thrombocytopenia (10.6%), dehydration (7.3%), and decreased platelet count (5.3%). The most common drug-related AEs observed with vorinostat in combination therapy (*n *= 157, most of whom received vorinostat 400 mg qd for 14 days) were nausea (48.4%), diarrhea (40.8%), fatigue (34.4%), vomiting (31.2%), and anorexia (20.4%), with the majority of AEs being Grade 2 or less. In Phase I trials, combinations with vorinostat were generally well tolerated and preliminary evidence of anticancer activity as monotherapy or in combination with other systemic therapies has been observed across a range of malignancies. Ongoing and planned studies will further evaluate the potential of vorinostat in combination therapy, including combinations with radiation, in patients with diverse malignancy types, including non-small-cell lung cancer, glioblastoma multiforme, multiple myeloma, and myelodysplastic syndrome.

## Histone Deacetylase Inhibition with Vorinostat as a Target in Oncology

Advanced or refractory malignancy remains an area of high unmet medical need as patients often relapse and curative therapy is elusive. The mainstay of treatment is generally cytotoxic chemotherapy which can have limited efficacy and is often associated with significant toxicity; there is a need for novel agents that are not only effective but also well tolerated. In particular, there has been increasing interest in targeted therapies which work at an epigenetic level to influence gene expression and ultimately control tumor growth and proliferation. Histone deacetylase (HDAC) inhibitors represent one such class of new mechanism-based anticancer drugs [1].

Modifications to histones influence chromatin structure, and ultimately gene transcription, including those coding for tumor suppressor proteins. One of the key histone modifications that controls gene transcription is acetylation, which is regulated by two opposing enzymatic activities (histone acetyltransferases [HATs] and HDACs) [1]. Histone acetylation leads to an open chromatin structure, and allows access to transcription binding sites. Although histones are one of the targets of HATs and HDACs, many nonhistone proteins, including transcription factors, tubulin and heat shock protein 90, can also be regulated by acetylation [2,3].

**Figure 1:**
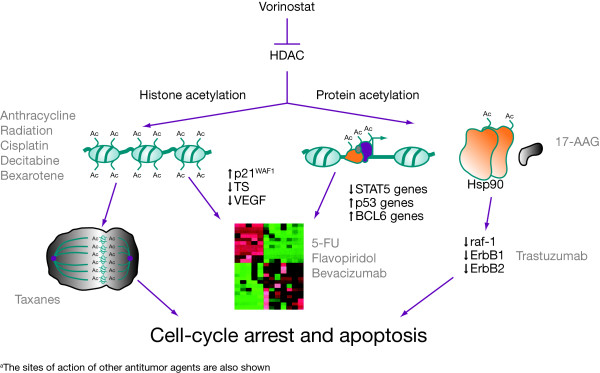
**Proposed mechanism of action of vorinostat in inducing tumor cell-cycle arrest and apoptosis^a ^**[4]. HDAC, histone deacetylase; TS, thymidylate synthase; VEGF, vascular endothelial growth factor; 17-AAG, 17-allylamino-17-demethoxygeldanamycin; 5-FU, 5-fluorouracil. Reprinted by permission from Macmillan Publishers Ltd: Richon VM. Cancer biology: mechanism of antitumour action of vorinostat (suberoylanilide hydroxamic acid), a novel histone deacetylase inhibitor. Br J Cancer 2006; 95 (Suppl 1): S2–S6, copyright 2006.

HDACs have been shown to be overexpressed in human cancers, such as gastric, prostate and colon cancer, and are involved in the regulation of transcription with recruitment by oncogenic transcription factors [4]. Therefore, the inhibition of HDACs is a rational target for the development of novel anticancer therapy. To date, 18 HDACs have been identified in mammalian cells, which are categorized into different classes, based on their homology to yeast deacetylases [5]. By inhibiting these enzymes, HDAC inhibitors permit chromatin to assume a more relaxed conformational state, thereby allowing transcription of genes involved in tumor suppression, cell-cycle arrest, cell differentiation, and apoptosis (Figure 1[4]) [6].

A variety of HDAC inhibitors are in clinical development and are being assessed in a number of different cancer indications [7]. There are several chemical families among the HDAC inhibitors, including short-chain fatty acids (butyrate, valproic acid), hydroxamates (vorinostat, trichostatin A, LBH-589, PXD-101), cyclic tetrapeptides (depsipeptide), and benzamides (MS-275, MGCD-0103). Vorinostat (Zolinza^®^; Merck & Co., Inc., Whitehouse Station, NJ, USA) was the first HDAC inhibitor licensed for clinical use and has been shown to inhibit the activity of class I and II HDACs, in particular HDAC1, HDAC2, HDAC3 (class I), and HDAC 6 (class II) at low nanomolar concentrations [4,5,8]. In addition to chromatin histone proteins that are involved in the regulation of gene expression, HDACs have many nonhistone protein targets including transcription factors and proteins that regulate cell proliferation, migration, and death [5]. For example, HDAC 6, which is predominantly cytosolic, has been shown to have roles in microtubule stability and function via the acetylation of α-tubulin [9], in the regulation of heat-shock protein 90 [10], and in the formation of aggresomes of ubiquitinylated proteins [11].

## Vorinostat Monotherapy for Solid and Hematologic Malignancies

Vorinostat is the first HDAC inhibitor approved for the treatment of cancer: in October 2006, the US Food and Drug Administration granted approval to vorinostat for the treatment of cutaneous manifestations of cutaneous T-cell lymphoma (CTCL) in patients with progressive, persistent or recurrent disease on or following two systemic therapies [12]. This approval was based on a pivotal Phase IIb multicenter trial of vorinostat monotherapy, which included 74 patients with persistent, progressive or recurrent, stage IB or higher CTCL who had received at least two prior systemic therapies including bexarotene [13]. The objective response rate was 30% and the most common drug-related adverse events (AEs) were diarrhea (49%), fatigue (46%), nausea (43%), and anorexia (26%). Most of these AEs were Grade 2 or lower but 21/74 patients (28%) had drug-related Grade 3/4 AEs, the most common being fatigue (5%), pulmonary embolism (5%), thrombocytopenia (5%), and nausea (4%). Similar results were observed in a second, smaller Phase II study including 33 patients with CTCL who were refractory to or intolerant of conventional therapy [14]. In this study, 8/33 patients (24%) achieved a partial response and the most common drug-related AEs were fatigue (73%), thrombocytopenia (54%), diarrhea (49%), nausea (49%), dysgeusia (46%), dry mouth (35%), and weight loss (27%). The most common drug-related Grade 3 or 4 AEs were thrombocytopenia (19%) and dehydration (8%). Overall, these studies showed that vorinostat as monotherapy was effective in advanced CTCL and had an acceptable safety profile. Vorinostat is included in the National Comprehensive Cancer Network Clinical Practice Guidelines in Oncology™ for non-Hodgkin's lymphoma (NHL), where it is listed as a systemic therapy option for patients with mycosis fungoides/Sézary syndrome who have failed multiple treatments with local and skin-directed therapy or who have unfavorable prognostic features [15].

Phase I studies have indicated that vorinostat monotherapy has an acceptable safety profile in patients with a variety of solid and hematologic malignancies [16-25]. Similarly, Phase II studies in patients with head and neck cancer [26], diffuse large B-cell lymphoma (DLBCL) [27], glioblastoma multiforme (GBM) [28], hormone-refractory prostate cancer [29], breast cancer [30], NHL [31], Hodgkin's lymphoma [32], non-small-cell lung cancer (NSCLC) [33], breast, colorectal or NSCLC [34], epithelial ovarian or primary peritoneal carcinoma [35], and myelodysplastic syndrome [36], have also shown that vorinostat is well tolerated, with preliminary activity as monotherapy against NHL and GBM [28,31].

In the Phase II study of vorinostat monotherapy in patients with GBM, 66 patients who had received ≤ 1 prior chemotherapy regimen for progressive/recurrent GBM, and who were not undergoing surgery, were treated with 200 mg vorinostat bid on Days 1–14 every 3 weeks [28]. The primary efficacy endpoint was met; nine of the first 52 patients were progression-free at 6 months, and the median overall survival was 5.7 months. As in the earlier CTCL studies, the majority of AEs were Grade 2 or lower; the most common Grade 3 or 4 AEs were thrombocytopenia (22%), fatigue (17%), neutropenia (8%), dehydration (6%), and hypernatremia (5%). In a subgroup of five patients with surgical recurrent GBM who received vorinostat prior to surgery, immunohistochemical analysis of paired baseline and post-vorinostat samples showed increased acetylation levels of histones H2B and H4, and histone H3 following vorinostat therapy in four of five and three of five patients, respectively. Microarray analysis of RNA extracted from the same paired samples revealed changes in the expression pattern of genes regulated by vorinostat, such as upregulation of E-cadherin (p = 0.02). These results suggest that the dose and schedule of vorinostat employed in this Phase II trial had a biologic effect on glioblastoma tumors, affecting target pathways in GBM. The authors of this study concluded that vorinostat has single-agent activity in GBM and is well tolerated.

In the other Phase II monotherapy study that demonstrated preliminary clinical activity, of 37 enrolled patients with relapsed or refractory follicular, marginal zone or mantle cell lymphoma, five patients achieved a complete response and five a partial response [31].

While there has not been clear evidence of QTc prolongation due to vorinostat in either preclinical or clinical studies to date, isolated clinical events of QTc prolongation in previous vorinostat studies have been observed, and QTc prolongation has been reported for other HDAC inhibitors [37,38]. However, in a Phase I randomized, placebo-controlled, crossover study conducted in 25 patients with relapsed or refractory advanced cancer, administration of a single supratherapeutic dose of vorinostat (800 mg) did not prolong the QTcF interval (monitored over 24 hours) [39]. The upper limit of the 90% confidence interval for the placebo-adjusted mean change-from-baseline of vorinostat was less than 10 ms at every time point for all 24 patients included in the QTcF analysis. For the vorinostat and placebo groups, there were no observed QTcF changes from baseline values >30 ms and only one patient experienced a QTcF interval >450 ms (seen following both vorinostat and placebo administration).

The acceptable safety profile of vorinostat observed in these studies, together with the monotherapy activity in some tumor types, provide a good foundation for the use of vorinostat in combination regimens.

## Biologic Rationale for Vorinostat Use in Combination with Other Therapies

Combination chemotherapy or chemoradiotherapy are frequently employed in preference to single-agent therapy to maximize treatment efficacy, but can be associated with increased toxicity. Vorinostat has a different mechanism of action compared with many other antineoplastic agents; therefore, it may be able to improve clinical efficacy in combination with other systemic agents where there are no or minimal overlapping toxicities. In addition, it has been hypothesized that the mechanism of action of HDAC inhibitors, through the acetylation of key lysine residues in core histones leading to a more relaxed chromatin configuration, may allow enhanced access to the DNA by another antineoplastic agent that directly interacts with DNA (e.g. cisplatin) resulting in synergistic activity [40].

Combination strategies may also help to overcome potential mechanisms of drug resistance to HDAC inhibitors [41]. These include other chromatin alterations such as DNA methylation, which together with hypoacetylation is thought to cooperate to induce gene silencing. Thus, the combination of HDAC inhibitors with hypomethylating agents, such as azacitidine and decitabine, is rational. Any protection against the cellular oxidative stress induced by HDAC inhibitors, such as proteins that participate in the stress response to oxidative damage, has also been postulated as a mechanism of resistance to HDAC inhibitors. In this case, the combination of HDAC inhibitors with other agents that also induce oxidative damage, such as bortezomib or doxorubicin, could help to overwhelm the stress response.

Numerous preclinical studies of vorinostat in combination with other cancer therapies have demonstrated synergistic or additive activity in cell lines from a wide range of solid and hematologic malignancies [4,5], including NSCLC [42-46], multiple myeloma (MM) [47-49], and leukemia [45,50-61]). In various models, treatment with vorinostat in combination resulted in synergistic apoptotic effects with associated increases in reactive oxygen species and mitochondrial injury, caspase and poly (ADP-ribose) polymerase activation. Synergistic activity has also been demonstrated *in vivo; *in one study in orthotopic human pancreatic tumors, the addition of vorinostat to bortezomib, and the resulting inhibition of HDAC 6 and disruption of aggresome formation, led to much higher levels of apoptosis and significantly reduced pancreatic tumor weight compared with either agent alone [62].

Some preclinical data also indicate that the activity of vorinostat in combination with radiation may be promising [63-66]. Vorinostat is to be tested in the adjuvant setting of GBM in combination with radiotherapy and temozolomide [67], and further trials are ongoing or planned in brain metastases and other indications where radiotherapy is used alone and in combination.

On the basis of these and other studies, vorinostat in combination is being evaluated in clinical trials in patients with a variety of solid and hematologic malignancies.

## Vorinostat in Combination for Advanced Solid Tumors

A number of Phase I studies have been undertaken to determine the recommended Phase II dose of vorinostat in combination with other established chemotherapy agents in patients with advanced or refractory solid tumors [68-74] (Table 1[68-74]). In one of these studies, in which vorinostat was combined with carboplatin and paclitaxel, particularly promising activity was noted in patients with advanced NSCLC, with 10/19 patients (53%; 18 chemonaïve) experiencing a partial response and 4/19 (21%) stable disease [68]. In comparison, treatment with carboplatin-paclitaxel of chemonaïve patients with advanced NSCLC results in response rates of approximately 15–25% [75-77]. The combination was generally well tolerated. Grade 3/4 toxicity was predominantly hematologic: of 28 treated patients, 2 patients experienced Grade 4 febrile neutropenia, and 8 and 14 patients experienced Grade 3 and 4 neutropenia, respectively; although this was more than expected from carboplatin-paclitaxel alone, with rates of Grade 4 neutropenia of 17–43% previously reported [75-77], there was no definite relationship found between the dose and schedule of vorinostat and the incidence of Grade 3/4 neutropenia. Dose-limiting toxicities (DLTs) were Grade 3 vomiting (one patient) and Grade 4 febrile neutropenia (one patient) and the recommended Phase II dose for vorinostat in combination with carboplatin-paclitaxel was 400 mg qd for 14 days every 3 weeks. In another study, vorinostat was combined with doxorubicin without exacerbation of doxorubicin toxicity, with a tolerated vorinostat dose of 400 mg bid dosed on Days 1–3 every week [71].

**Table 1:** Phase I Results of Vorinostat in Combination Therapy in Patients with Advanced Solid Tumors

**Tumor Type**	**No. Pts**	**Treatment**	**Summary of Results**	**Ref**
Advanced solid	22	Vorinostat + pemetrexed + cisplatin	DLTs: fatigue (2), dehydration (2), neutropenia (1), cerebral ischemia (1) DVT (1) 19 patients evaluable for response: 1 CR, 1 PR, 11 SD, 6 PD Vorinostat 300 mg qd for 7/21 days was tolerable with cisplatin 75 mg/m^2 ^+ pemetrexed 500 mg/m^2^	[70]
Advanced solid	20	Vorinostat + doxorubicin	DLTs: thrombocytopenia (1), fatigue (1), nausea/vomiting, and anorexia (1) Response: 1 PR, 3 SD, 11 PD, 5 NE Tolerated dose of vorinostat higher than approved single-agent dose in patients with hematologic malignancies	[71]
Advanced colorectal	21	Vorinostat + 5-FU/LV + oxaliplatin	DLTs: fatigue (1), fatigue and diarrhea (1), fatigue, anorexia, and dehydration (1) Response: 11 SD (5 confirmed) of 21 evaluable patients Recommended dose: vorinostat 300 mg bid on Days 1–7 + 5-FU/LV + oxaliplatin on Day 4 every 14 days	[74]
Advanced solid	28	Vorinostat + carboplatin + paclitaxel	DLTs: vomiting (1), febrile neutropenia (1) Response: 11 PR, 7 SD in 25 evaluable patients (of 19 pts with NSCLC [18 chemonaïve], 10 [53%] had a PR) Phase II regimen: vorinostat 400 mg qd on Days 1–14 + carboplatin AUC 6 mg/mL × min + paclitaxel 200 mg/m^2^	[68]
Refractory solid	22	Vorinostat + bortezomib	DLTs: fatigue (3), hyponatremia (1), elevated ALT (1) MTD (step A): vorinostat 400 mg qd on Days 1–14 + bortezomib 1.3 mg/m^2 ^on Days 1, 4, 8, and 11 of a 21-day cycle Clinical activity observed: 1 PR >9 months in a patient with refractory soft tissue sarcoma	[72]
Advanced solid	26	Vorinostat + capecitabine	DLTs: diarrhea (1), fatigue (2), nausea/vomiting (1) Response: 4 PR (3 confirmed), 18 SD, 4 PD Recommended Phase II regimen: vorinostat 300 mg qd + capecitabine 1000 mg/m^2 ^bid	[73]
Malignant glioma	19	Vorinostat + temozolomide	DLTs: thrombocytopenia (2), fatigue (3), nausea (1) MTD: vorinostat 300 mg qd on Days 1–14 + temozolomide 150 mg/m^2^/day on Days 1–5 every 28 days	[69]

DLT, dose-limiting toxicity; ALT, alanine aminotransferase; MTD, maximum tolerated dose; PR, partial response; DVT, deep vein thrombosis; CR, complete response; PR, partial response; SD, stable disease; PD, disease progression; NE, not evaluable; NSCLC, non-small-cell lung cancer; AUC, area under the curve; 5-FU/LV, 5-fluorouracil/leucovorin.

The results of disease-specific Phase I vorinostat combination studies in patients with malignant gliomas [69] or colorectal cancer [74] have also been published (Table 1[68-74]). In patients with malignant gliomas treated with escalating doses of vorinostat plus temozolomide, DLTs were Grade 3 thrombocytopenia, Grade 3 nausea, and Grade 4 thrombocytopenia each reported in one patient, and Grade 3 fatigue reported in three patients [69]. The recommended Phase II dose for vorinostat in combination with temozolomide was 300 mg qd on Days 1–14 every 28 days.

Overall, the data of vorinostat in combination regimens for the treatment of a variety of advanced solid tumors demonstrate that, when used with other chemotherapy agents, vorinostat can be well tolerated and the preliminary anticancer activity noted supports the conduct of disease-specific Phase II studies. A range of ongoing studies will further evaluate the role of vorinostat in combination therapy in a variety of advanced solid tumors; these include Phase I/II studies with vorinostat in combination in patients with advanced breast cancer, small-cell lung cancer, and NSCLC, and Phase II studies in combination with tamoxifen or carboplatin and paclitaxel in patients with advanced breast cancer or in combination with carboplatin and paclitaxel in patients with advanced NSCLC [67].

## Vorinostat in Combination for Hematologic Malignancies

Vorinostat also has potential in combination with chemotherapy or other biologic agents as treatment for hematologic malignancies. The combination of vorinostat plus the proteasome inhibitor bortezomib has been investigated in two Phase I studies in heavily pretreated patients with advanced relapsed or refractory MM [78,79] (Table 2[78-87]). In one of these studies, one patient receiving vorinostat 400 mg qd on Days 1–14 plus bortezomib 0.9 mg/m^2 ^on Days 1, 4, 8, and 11 every 21 days experienced a DLT of Grade 3 transient aspartate aminotransferase elevation and one patient receiving vorinostat 400 mg qd plus bortezomib 1.3 mg/m^2 ^experienced a DLT of Grade 4 thrombocytopenia [79]. The most common (≥ 10% of patients) Grade 3/4 drug-related AEs were thrombocytopenia (38%) and fatigue (12%). Dose escalation was successfully completed and the maximum tolerated dose (MTD) was not reached. The maximum administered dose was vorinostat 400 mg qd on Days 1–14 plus bortezomib 1.3 mg/m^2 ^on Days 1, 4, 8, and 11 every 21 days. In the second of these studies, MTD was established at 400 mg qd on Days 4–11 plus bortezomib 1.3 mg/m^2 ^on Days 1, 4, 8, and 11 every 21 days, with DLTs of Grade 3 prolonged QT interval and Grade 3 fatigue each reported in one patient [78].

**Table 2:** Phase I Results of Vorinostat in Combination Therapy in Patients With Hematologic Malignancies^a^

**Tumor Type**	**No. Pts**	**Treatment**	**Summary of Results**	**Ref**
Relapsed multiple myeloma	23	Vorinostat + bortezomib	DLTs: prolonged QT interval (1), fatigue (1) MTD vorinostat 400 mg qd on Days 4–11 + bortezomib 1.3 mg/m^2 ^on Days 1, 4, 8, and 11 every 21 days Response: 2 VGPR, 7 PR, 10 SD (21 evaluable patients)	[78]
Relapsed, refractory or poor prognosis acute leukemia or refractory anemia with excess blasts-2	22	Vorinostat + flavopiridol (bolus or 'hybrid' infusion schedules)	DLTs: infectious colitis with sepsis (1 [bolus]) and atrial fibrillation (1 ['hybrid']) MTD: not yet reached on vorinostat 200 mg tid given in a 'hybrid' schedule with flavopiridol at 30/30 mg/m^2 ^(load/infusion) on Days 1 and 8 of a 21-day cycle, identification of the MTD and recommended phase II dose is ongoing Response: 10 patients experienced some clinical benefit (20 evaluable patients)	[81]
Advanced acute leukemia	20	Vorinostat + idarubicin	DLTs: myelosuppression, encephalopathy, and dysphagia 2 CR and 2 complete marrow responses observed in patients who had failed previous anthracycline-based therapy Recruitment ongoing at vorinostat 400 mg tid for 3 days + idarubicin 12 mg/m^2 ^for 3 days every 14 days	[82]
Relapsed or newly-diagnosed acute myelogenous leukemia or myelodysplastic syndrome	70	Vorinostat + decitabine (concurrent or sequential regimens)	DLT: prolonged QT interval (1 [sequential]) Response: concurrent (n = 34), 7 CR, 2 PR, 2 HI, 12 SD; sequential (n = 36), 3 CR, 2 HI, 16 SD MTD not reached Last cohort: vorinostat 400 mg qd for 14 days (Days 1–14 concurrent or Days 6–19 sequential) + decitabine 20 mg/m^2^/day on Days 1–5 every 28 days	[83]
Relapsed, refractory or poor prognosis leukemia	31	Vorinostat + decitabine	DLTs: pulmonary embolism and diarrhea (1) Response: 1 CR, 4 significant reduction in bone marrow blasts, 4 SD, 14 PD, 7 NE (30 evaluable patients) Last cohort: decitabine 25 mg/m^2 ^daily for 5 days followed by vorinostat 200 mg tid for 14 days	[84]
Relapsed or refractory multiple myeloma	18	Vorinostat + lenalidomide + dexamethasone	DLTs: none yet reported MTD: not yet reached, DLT evaluation ongoing in patients enrolled to vorinostat 400 mg qd for 14 days (Days 1–7 and 15–21), combined with lenalidomide 25 mg qd for 21 days, and dexamethasone 40 mg/day (Days 1, 8, 15, and 22) every 28 days Response: 1 CR, 4 PR, 1 MR, 5 SD (15 evaluable patients)	[87]
Myelodysplastic syndrome and acute myeloid leukemia	28	Vorinostat + azacitidine	DLTs: not reported Response: 9 CR, 2 incomplete CR, 7 HI, 2 SD (21 evaluable patients) Last cohort: azacitidine 55 mg/m^2^/day on Days 1–7 + vorinostat 300 mg bid on Days 3–5 every 28 days	[85]
Advanced multiple myeloma	34	Vorinostat + bortezomib	DLTs: transient AST elevation (1), thrombocytopenia (1) MTD not yet reached, the maximum administered dose was vorinostat 400 mg qd on Days 1–14 + bortezomib 1.3 mg/m^2 ^on Days 1, 4, 8, and 11 every 21 days. Response: 12 PR, 6 MR, 13 SD (33 evaluable patients). In 17 evaluable patients who had received prior bortezomib therapy, 6 PR, 4 MR, 7 SD	[79]
Acute myeloid leukemia	27	Vorinostat + decitabine	DLT: fatigue (1) Response: 1 incomplete CR, 1 morphologic leukemia-free (without neutrophil recovery), 3 PR (25 evaluable patients) MTD not reached: maximum dose vorinostat 200 mg bid on Days 1–21 + decitabine 20 mg/m^2^/day on Days 1–5 every 28 days	[86]

^a^Only trials including at least 15 patients are reported in this table.

DLT, dose-limiting toxicity; AST, aspartate aminotransferase; MTD, maximum tolerated dose; PR, partial response; MR, minimal response; SD, stable disease; VGPR, very good partial response; nCR, near complete response; PD, progressive disease; CR, complete response; NE, not evaluable; HI, hematologic improvement.

Efficacy appeared to be similar in these two studies: in the first study, of 33 patients evaluable for efficacy, 12 had a partial response, 6 had a minimal response (overall 55% response), and 13 had stable disease; 2 patients experienced progressive disease [79]. In the second study, which included more heavily pretreated patients (median number of prior regimens 7 versus 3), 9/21 patients (43%) had a response, 10 had stable disease, and 2 had disease progression [78]. In contrast, only modest single-agent activity was observed with vorinostat in patients with relapsed/refractory MM, with 1/10 evaluable patients having a minimal response and 9/10 stable disease [25].

Preliminary data from Phase I studies have shown that vorinostat is well tolerated when combined with cytarabine and etoposide for the treatment of advanced acute leukemia and high-risk myelodysplastic syndrome [80], with flavopiridol in refractory or high-risk acute myeloid leukemia [81], or in combination with lenalidomide and dexamethasone in patients with relapsed or refractory MM [87]. Other ongoing Phase I studies of vorinostat combinations in patients with hematologic malignancies have also shown that combinations with idarubicin, decitabine or azacitidine are well tolerated [82-86] and have suggested potential anticancer activity of vorinostat in combination with idarubicin, in patients with advanced leukemia [82], decitabine, in patients with advanced leukemia [84], acute myeloid leukemia [83,86], or myelodysplastic syndrome [83], or azacitidine in patients with myelodysplastic syndrome or acute myeloid leukemia [85] (Table 2[78-87]). Again, the tolerability profile and preliminary anticancer activity support the continuing investigation of combinations of vorinostat with other chemotherapy agents in disease-specific Phase II studies. Ongoing clinical trials will further evaluate the role of vorinostat in combination therapy in hematologic malignancies, such as MM, leukemia, and lymphoma [67].

## Safety and Tolerability of Vorinostat – Overall Experience from the Vorinostat Clinical Trial Program

Analysis of combined safety data from the vorinostat clinical trial program of Phase I and II trials demonstrate that vorinostat has an acceptable safety and tolerability profile either as monotherapy or combination therapy in patients with a variety of solid and hematologic malignancies. At a cut-off date of April 2008, collated data were available for 341 patients who received vorinostat as monotherapy for either solid tumors (mesothelioma, head and neck, renal, thyroid, laryngeal, breast, colorectal, NSCLC, and gastric cancers) or for hematologic malignancies (acute myeloid leukemia, chronic lymphocytic leukemia, or chronic myeloid leukemia, NHL [including CTCL, peripheral T-cell lymphoma, DLBCL, and follicular lymphoma], Hodgkin's disease, myelodysplastic syndrome or MM). Of these patients, 156 patients were treated at a dose of 400 mg qd (the current FDA-approved dose for patients with CTCL). The most commonly reported drug-related AEs were fatigue (62%), nausea (56%), diarrhea (49%), anorexia (48%), and vomiting (33%) (Table 3). Grade 3/4 drug-related AEs included fatigue (12%), thrombocytopenia (11%), dehydration (7%), and decreased platelet count (5%). Three drug-related deaths (ischemic stroke, tumor hemorrhage, unspecified) were observed.

**Table 3:** Drug-Related Adverse Events Occurring in ≥ 15% of Patients Who Received Vorinostat Monotherapy in the Vorinostat Clinical Trial Program (Data Cut-Off April 2008)

**Adverse Event**	**No. (%) of Patients (*N *= 341)**	
	
	**All Grades**	**Grade 3 or 4**
Fatigue	211 (61.9)	41 (12.0)
Nausea	190 (55.7)	14 (4.1)
Diarrhea	168 (49.3)	14 (4.1)
Anorexia	164 (48.1)	17 (5.0)
Vomiting	112 (32.8)	5 (1.5)
Blood creatinine increased	88 (25.8)	2 (0.6)
Weight decreased	86 (25.2)	4 (1.2)
Hyperglycemia	79 (23.2)	10 (2.9)
Thrombocytopenia	71 (20.8)	36 (10.6)
Platelet count decreased	65 (19.1)	18 (5.3)
Hemoglobin decreased	60 (17.6)	10 (2.9)
Constipation	60 (17.6)	3 (0.9)
Dysgeusia	59 (17.3)	0 (0.0)

Similarly, collated safety data from 157 patients who received vorinostat (most commonly at 400 mg qd for 14 days) in combination with other systemic therapies in the vorinostat clinical trial program were available for analysis (cut-off date of April 2008). Patients received vorinostat in combination with other systemic therapies for the treatment of advanced cancer, MM, CTCL, and NSCLC. In combination, the most commonly reported drug-related AEs were nausea (48%), diarrhea (41%), fatigue (34%), vomiting (31%), and anorexia (20%) (Table 4). The most common Grade 3/4 events were fatigue (13%), thrombocytopenia (10%), neutropenia (8%), diarrhea (6%), and nausea (5%). There was one drug-related AE leading to death due to hemoptysis in one patient with NSCLC.

**Table 4:** Drug-Related Adverse Events Reported by ≥ 15% of Patients Who Received Vorinostat Combination Therapy in the Vorinostat Clinical Trial Program (Data Cut-Off April 2008)

**Adverse Event**	**No. (%) of Patients (*N *= 157)**	
	
	**All Grades**	**Grade 3 or 4**
Nausea	76 (48.4)	8 (5.1)
Diarrhea	64 (40.8)	9 (5.7)
Fatigue	54 (34.4)	21 (13.4)
Vomiting	49 (31.2)	6 (3.8)
Anorexia	32 (20.4)	4 (2.5)
Dehydration	28 (17.8)	6 (3.8)
Thrombocytopenia	25 (15.9)	15 (9.6)
Anemia	25 (15.9)	4 (2.5)

Overall, vorinostat was well tolerated, with the majority of AEs being Grade 2 or less, and vorinostat was not associated with the levels of hematologic toxicity commonly found with other antineoplastic agents. Furthermore, dose modifications were usually not required in the majority of patients who received vorinostat as monotherapy or in combination therapy.

## Conclusion

Vorinostat is generally well tolerated and has shown potential anticancer activity against a variety of hematologic and solid tumors, particularly in combination therapy, as well as in monotherapy. As monotherapy, combined data from the vorinostat clinical trial program demonstrate that vorinostat has an acceptable safety and tolerability profile, with the most common Grade 3/4 AEs being fatigue (12%) and thrombocytopenia (11%). Although the tolerability data from Phase I trials of vorinostat in combination are limited, the individual trial data suggest that the combinations are also generally well tolerated, and this appears to be substantiated by pooled safety data from the vorinostat clinical trial program. Despite concerns, the available data suggest that there do not appear to be any unexpected toxicities when vorinostat is combined with other antineoplastic agents. These preliminary clinical results from Phase I and II trials support the rationale for combining vorinostat with other chemotherapy agents and/or radiotherapy as a means of increasing the therapeutic index of cancer therapy.

## Competing interests

SR, JGV, and CSR are employees of Merck & Co., Inc. VMR was a founder of Aton Pharma Inc. and an employee of Merck & Co., Inc., and is now employed by EpiZyme Inc. MH is now employed by the Celgene Corporation.

Merck employees may own shares or stock options of Merck & Co., Inc. CB is a consultant for Merck & Co., Inc. DS, EG, and FR have no relevant financial disclosures to declare.

## Authors' contributions

All authors (DS, MH, CB, FR, EG, VMR, SR, JGV and CSR) participated in drafting and editing the manuscript and all authors read and approved the final manuscript.
